# Laparoscopic Duodenum and Spleen-Preserving Subtotal or Total Pancreatectomy: A Parenchyma-Sparing Strategy for Main Duct Intraductal Papillary Mucinous Neoplasms (with Video)

**DOI:** 10.1245/s10434-024-16244-2

**Published:** 2024-09-23

**Authors:** Zheng Li, Yihua Shi, Wenjie Tang, Chen Chen, Wensheng Liu, Qifeng Zhuo, Shunrong Ji, Chenjie Zhou, Xianjun Yu, Xiaowu Xu

**Affiliations:** 1 Department of Pancreatic Surgery, Fudan University Shanghai Cancer Center, Shanghai, China; 2 Department of Oncology, Shanghai Medical College, Fudan University, Shanghai, China; 3 Shanghai Pancreatic Cancer Institute, Shanghai, China; 4 Pancreatic Cancer Institute, Fudan University, Shanghai, China; 5 Department of Nursing Administration, Fudan University Shanghai Cancer Center, Shanghai, China

**Keywords:** Intraductal papillary mucinous neoplasms, Main pancreatic duct, Laparoscopic surgical technique, Duodenum and spleen-preserving pancreatectomy, Prognosis

## Abstract

**Background:**

For premalignant main duct intraductal papillary mucinous neoplasms (MD-IPMN), laparoscopic duodenum and spleen-preserving subtotal or total pancreatectomy (LDSP-STP/TP) seems to be a viable option for parenchyma-sparing pancreatectomy.

**Patients and Methods:**

On the basis of the imaging features, family history, genomic alterations, intraoperative ultrasound examination, and frozen section evaluation, we have proposed patient selection strategies for the LDSP-STP/TP technique for the first time. Additionally, a comprehensive step-by-step overview of this technique has been provided. To date, we have performed five LDSP-STP procedures and one LDSP-TP procedure.

**Results:**

We successfully performed selective resection of the affected pancreatic parenchyma while preserving the duodenum, common bile duct (CBD), spleen, and splenic artery and vein. The operation time ranged from 295 to 495 min, with blood loss ranging from 100 to 300 mL. Postoperative pathological results revealed low-grade dysplasia in the resected pancreatic samples and margins. The patients resumed eating within 3–5 days after surgery, and all postoperative complications were classified as grade I according to the Clavien–Dindo classification. At the 3-month follow-up, there were no cases of CBD ischemic stenosis, splenic ischemia, or pseudocyst formation observed. For patients who received LDSP-STP, the longitudinal diameter of the remaining pancreatic tail ranged from 2.2 to 4.6 cm, and they demonstrated satisfactory long-term blood glycemic control.

**Conclusions:**

LDSP-STP/TP demonstrates technical feasibility and safety. It allows for the selective resection of the affected pancreatic parenchyma, thereby minimizing the impact of pancreatic functional impairment. However, it is crucial to validate this technique through long-term prospective observations.

**Supplementary Information:**

The online version contains supplementary material available at 10.1245/s10434-024-16244-2.

Intraductal papillary mucinous neoplasm (IPMN) is a significant precursor of pancreatic cancer.^1^ With the expanding use of high-quality cross-sectional imaging, the detection rate of IPMNs has significantly increased.^2,3^ However, there is still ongoing controversy regarding the assessment of their malignant potential and the selection of surgical approaches.^4,5^

In the current IPMN management guidelines, the indications for parenchyma-sparing pancreatectomy are limited. It is recommended only for patients without risk factors who express a strong desire for surgery. Patients who meet the surgical criteria should undergo oncological resection with standard lymphadenectomy. Prior to meeting these criteria, patients should undergo regular monitoring until they become unsuitable for surgery.^6^ Thus, patients with IPMN are confronted with the dilemma of lifelong follow-up until the tumor progresses to a stage necessitating oncological resection.^7^ Furthermore, the risk of malignancy transformation in IPMN increases gradually with age.^8^

As a prophylactic surgery, minimally invasive parenchyma-sparing pancreatectomy can effectively remove pancreatic lesions with potential malignant transformation risk while preserving pancreatic function to the greatest extent.^9^ Although postoperative follow-up is necessary for patients with IPMN, this approach eliminates the need for oncological resection and provides excellent long-term outcomes.^10^ Additionally, the rate of surgery-related complications is deemed acceptable.^11^

For patients with premalignant main duct IPMN (MD-IPMN), laparoscopic duodenum and spleen-preserving subtotal or total pancreatectomy (LDSP-STP/TP) is a viable option for parenchyma-sparing pancreatectomy. This multimedia article is the first to provide patient selection strategies, surgical procedures and prognosis for this technique.

## Patients and Methods

### Patient Selection Strategies

The current controversy surrounding the adoption of parenchyma-sparing pancreatectomy for MD-IPMN is primarily owing to the potential presence of early-stage cancer within the lesions. Therefore, the selection of indications for LDSP-STP/TP should be approached with caution, aiming to avoid over-treatment of patients who can be regularly monitored while also preventing the residual presence of early-stage cancer.

The patient selection strategies for LDSP-STP/TP in MD-IPMN are as follows:

(1) Presence of one or more worrisome features, such as pancreatitis, increased CA19-9 serum level, cyst ≥ 30 mm, enhancing mural nodule < 5 mm, thickened/enhancing cyst walls, main pancreatic duct (MPD) ≥ 5 mm and < 10 mm, change in caliber of pancreatic duct, lymphadenopathy, and cystic growth rate ≥ 2.5 mm/year.^6,8^ (2) No high-risk stigmata according to IPMN management guidelines, including obstructive jaundice; an enhancing mural nodule ≥ 5 mm or solid component; MPD ≥ 10 mm; suspicious or positive results of cytology (if performed); and new-onset or worsening diabetes.^6,8^ (3) No family history of pancreatic cancer; absence of germline mutations, such as BRCA2, BRCA1, PALB2, CDKN2A, ATM, and TP53, and mismatch repair genes MLH1, MSH2, and MSH6;^12^ the next-generation sequencing of pancreatic cyst fluid did not detect mutations in TP53, SMAD4, CTTNB1, mTOR, PIK3CA, PTEN, and/or AKT1 (if performed).^13,14^ (4) If the diameter of the MPD in the pancreatic tail was < 5 mm, evaluation for LDSP-STP can be considered. Intraoperative ultrasound is used to help determine where to transect the pancreas and provide more tumor imaging characterization information. (5) Intraoperative frozen section examination of the samples and resected pancreatic margin should be routinely performed. If high-grade dysplasia is detected, the STP approach should be converted to TP. If invasive carcinoma is detected, the surgery should be converted to oncological resection.

If the equipment conditions permit, the intraoperative pancreatoscope, along with forceps biopsy, can be utilized to evaluate the extent of involvement of the MPD and determine the type and scope of pancreatic resection.^15^ Moreover, it enables the detection of skip lesions during surgery for MD-IPMN.^16–18^

### Surgical Procedures

The LDSP-TP procedure is more challenging technically compared with LDSP-STP, we have provided a detailed description of the surgical procedure in Supplementary Video 1. First, position the patient in a supine posture, with the legs spread apart and in the anti-Trendelenburg position (30°). The “five-hole method” is utilized for the insertion of trocars. The surgical procedure is conducted using a three-dimensional laparoscopic system and maintaining a constant pressure pneumoperitoneum of 14 mmHg.

After opening the gastrocolic ligament, the stomach is suspended using laparoscopic gauze. Employ the Kimura technique to perform spleen-preserving subtotal or total resection of the pancreatic body and tail.^19^ The pancreatic capsule is incised along the inferior border of the pancreas toward the splenic hilum. The posterior pancreatic space is adequately dissected along the “fusion fascia of Toldt,” ligating and dividing any encountered dorsal pancreatic veins (DPV). The superior mesenteric vein (SMV) is dissected along the inferior border of the pancreatic neck, and the dorsal pancreatic artery (DPA) is ligated and divided. The sheath of the splenic vein is incised longitudinally using the ultrasonic scalpel, and small branches of the splenic vein are cauterized if necessary.

For patients with no dilation of the pancreatic duct in the tail of the pancreas, a subtotal pancreatectomy can be considered. Intraoperative ultrasound and pancreatoscopy can be used to assist in locating the site for pancreatic transection. Linear staplers are used to divide the pancreatic tissue, and the pancreatic margin is sent for intraoperative frozen section examination to ensure the absence of high-grade dysplasia or invasive cancer. The pancreatic tail remnant is reinforced with interrupted sutures. For patients without preservation of pancreatic parenchyma, the pancreatic tail is completely dissected from the splenic hilum using a “tail-first” approach, achieving an anatomical dissection of the pancreatic neck, body, and tail while preserving the splenic vessels.

Next, the duodenum-preserving pancreatic head resection (DPPHR) is performed using the technique first described by Beger.^20^ Ligating and dividing the gastrocolic trunk, the uncinate process is flipped toward the ventral and cephalic sides of the body, allowing access to the “fusion fascia of Treitz” plane using the ultrasonic scalpel. Histologically, the fusion fascia is composed of a loose connective tissue membrane, within which all the crucial pancreaticoduodenal arcades of arteries and veins are situated, between the membrane and the pancreatic parenchyma. Preserving the pancreaticoduodenal vascular arcades is of utmost importance in the DPPHR procedure to prevent serious complications, such as duodenal ischemic necrosis and bile duct ischemic stenosis. Ideally, the goal is to preserve the anterior and posterior vascular arcades intact. However, in the actual surgical process, anatomical variations in the anterosuperior pancreatic duodenal artery (ASPDA) are commonly encountered, often coursing within the pancreatic parenchyma of the head. Therefore, preserving the ASPDA can be challenging, and if necessary, it may need to be ligated and divided.

MD-IPMN often presents with concomitant chronic inflammation, resulting in a hardened pancreatic parenchyma and adhesions to surrounding tissues, leading to a frequently bloody surgical field during DPPHR. It is crucial to promptly irrigate and control bleeding. In cases with mild inflammatory response, the assistant can provide appropriate tension to clearly expose the anterior inferior pancreaticoduodenal artery (AIPDA) between the lower border of the pancreas head and the horizontal segment of the duodenum. Then, by dissecting closely along the pancreatic capsule towards the head, the posterior inferior pancreaticoduodenal artery (PIPDA) becomes visible. In some patients, the PIPDA may share a common trunk with the AIPDA, while in others, two separate PIPDAs may arise from the superior mesenteric artery (SMA). Once the course of the PIPDA is exposed, the dissection behind the head of the pancreas becomes easier, simply by dissecting along the pancreatic capsule and preserving the intact posterior pancreatic capsule. Further dissection toward the inner aspect of the posterior wall of the duodenum reveals the posterosuperior pancreaticoduodenal artery (PSPDA) and the posterior superior pancreaticoduodenal vein (PSPDV). The PSPDV is relatively larger and often collects two or more veins draining the head of the pancreas. Caution must be exercised when handling these veins to avoid significant bleeding that may inadvertently injure the PSPDA. Simultaneously, preserving the PSPDV is crucial to prevent postoperative duodenal congestion and edema. The PSPDA is the primary arterial supply to the pancreatic portion of the common bile duct (CBD), making its preservation particularly important. Additionally, the PSPDA gives rise to one or two arteries that extend toward the papilla of Vater, coursing along the right side of the CBD (the artery toward the papilla of Vater). Care must be taken to avoid injuring this artery during pancreatic resection.^21^

The CBD can be exposed starting from the ampulla or at the angle between the gastroduodenal artery (GDA) and the portal vein, as these locations have relatively fixed positions. The use of indocyanine green (ICG)-enhanced fluorescence imaging system can assist in visualizing the bile duct and its variations.^22^ Preoperative placement of a nasobiliary tube or biliary stent can also aid in exposing the CBD and managing early bile leaks. However, this procedure may lead to significant inflammation and edema of the pancreatic head tissue, posing great challenges during surgery, and it is recommended to be performed in experienced centers. Between the PSPDA and the CBD, there is often a portion of pancreatic tissue. Therefore, most surgeons recommend preserving this portion of pancreatic tissue to protect the vasculature from the PSPDA to the CBD. However, there are also surgeons who suggest complete removal of this portion of pancreatic tissue. During the dissection of the CBD, it is important to avoid clamping to prevent postoperative bile leakage. If there are concerns regarding poor blood supply or morphology of the CBD, or potential clamping or thermal injury, prophylactic T-tube placement can be considered to prevent postoperative bile leakage or CBD stricture. Cholangiography through the T-tube is performed one month postoperatively to confirm the absence of bile leaks or biliary strictures before T-tube removal. Ligating and dividing the MPD/secondary pancreatic duct, the pancreatic duct margin is sent for intraoperative frozen section examination. In some patients, there may not be a distinct gap between the inner wall of the descending portion of the duodenum and the pancreatic head tissue. Often, there is an evident disruption of the muscular layer of the duodenum after dissecting the pancreatic head. For such injuries, direct suturing and repair are usually sufficient.

### Perioperative Outcomes and Prognosis

Our surgical team has successfully performed six cases of LDSP-STP and one case of LDSP-TP for MD-IPMN in 2023, following a meticulous evaluation and selection process. Among these cases, one patient had intraoperative frozen section examination revealing high-grade dysplasia and focal carcinoma, necessitating a conversion to oncological resection. The clinicopathological characteristics and prognosis of the remaining six patients can be found in Table 1.

**Table 1:** Clinicopathological characteristics and prognosis

Patient	No. 1	No. 2	No. 3	No. 4	No. 5	No. 6
Age, years	66	67	59	72	69	61
Pancreatitis	No	Yes	Yes	No	No	Yes
Diabetes	No	No	Yes	No	No	Yes
CA19-9, U/mL	6.5	30.0	2.0	15.4	2.0	23.0
MPD diameter, mm	6.0	8.5	8.0	6.5	6.0	8.2
ASA grade	II	II	II	II	II	II
Pancreatic parenchymal resection extent	Subtotal	Subtotal	Total	Subtotal	Subtotal	Subtotal
T-tube drainage	No	No	Yes	Yes	Yes	No
Duodenal ischemia	No	No	No	No	No	No
Operation time, minutes	366	495	423	296	305	295
Blood loss, mL	100	100	300	200	200	200
Dysplasia grade	Low	Low	Low	Low	Low	Low
Postoperative LOS, days	12	10	10	17	11	10
Clavien-Dindo classification	I	I	I	I	I	I
POPF grade	Biochemical	B	Biochemical	B	B	B
Postoperative hemorrhage	No	No	No	No	No	No
Bile leakage	No	No	No	No	No	No
Delayed gastric emptying	No	No	No	No	No	No
Time to resume eating, days	4	4	3	3	4	5
*Postoperative follow-up after 3 months*						
Residual pancreatic tail length, cm	2.2	4.6	0.0	2.6	3.0	3.9
CBD ischemic stenosis	No	No	No	No	No	No
Splenic ischemia	No	No	No	No	No	No
Pseudocyst	No	No	No	No	No	No
Fasting blood glucose, mmol/L	5.0	6.0	6.0	6.0	6.3	7.0
C-peptide, nmol/L	0.7	0.63	0.1	0.2	0.4	0.1
Insulin, pmol/L	42.1	26.3	2.9	15.5	106	3.6
HbA1c, %	6.7	5.7	8.0	6.4	5.7	5.3

*CA19-9* carbohydrate antigens 19-9, *MPD* main pancreatic duct, *ASA* American Society of Anesthesiologists, *LOS* length of stay, *POPF* postoperative pancreatic fistula, *CBD* common bile duct, *HbA1c* hemoglobin A1c

The age of the patients ranged from 59 to 72 years, with three patients having a history of pancreatitis and two patients with a history of diabetes. The serum CA19-9 levels of all patients were within the normal range. Preoperative imaging evaluations revealed a maximum diameter of the MPD ranging from 6.0 to 8.5 mm. Among the patients, five underwent LDSP-STP, while one patient received LDSP-TP due to extensive dilation of the MPD. As a preventive measure against postoperative CBD fistula, three patients had a prophylactic placement of a T-tube. Prior to abdominal closure, all patients demonstrated good blood supply to the duodenum and spleen without any signs of ischemia. The operation time ranged from 295 to 495 min, with blood loss ranging from 100 to 300 mL. Postoperative pathological results revealed low-grade dysplasia in the resected pancreatic samples and margins.

The patients resumed eating within 3–5 days after surgery. The postoperative length of stay ranged from 10 to 17 days, and the Clavien–Dindo classification of postoperative complications was grade I in all patients. Four patients experienced grade B postoperative pancreatic fistula owing to prolonged drainage tube placement exceeding 3 weeks. Additionally, the prophylactic postoperative use of octreotide, no interventional treatments, such as abdominal puncture drainage, were employed. No patients experienced postoperative hemorrhage, bile leakage, or delayed gastric emptying.

At the 3-month follow-up, patients underwent surveillance and re-examination. Enhanced computed tomography scans revealed a longitudinal diameter of the pancreatic tail ranging from 2.2 to 4.6 cm. No CBD ischemic stenosis, splenic ischemia, or pseudocyst formation was observed. Among the six patients, patient no. 5 did not require medication to control blood glucose levels, while the remaining five managed their levels through subcutaneous insulin injections. The fasting blood glucose levels ranged from 5.0 to 7.0 mmol/L. Except for two patients (no. 3 and 6) who had pre-existing diabetes, three out of the remaining four patients had normal fasting C-peptide and insulin levels. Only the patient (no. 3) who underwent LDSP-TP had poor long-term blood glycemic control, as indicated by an HbA1c level of 8.0%.

## Discussion

A comprehensive understanding of the local anatomy of the pancreatic head contributes to the promotion and improvement of the DPPHR technique.^20^ Kimura et al. conducted a meticulous examination of the anatomy of the pancreas head and duodenum using materials from 40 autopsy cases. They proposed that it may be possible to remove the head of the pancreas while preserving the vascular arcades and their branches to the duodenum, the CBD, and the papilla of Vater.^23^ Building upon the DPPHR technique, the Liverpool procedure, known as duodenum and spleen-preserving total pancreatectomy (DSP-TP), has been developed and can be utilized for treating end-stage chronic pancreatitis and chronic abdominal pain. However, owing to the inflammatory process causing dense adherence of both the splenic artery and vein to the pancreas, the authors employed the Warshaw technique to resect the splenic artery and vein^24,25^

For the treatment of benign or low-grade malignant neoplasms that are spreading throughout the entire pancreas, the most ideal approach is to selectively remove the affected pancreatic parenchyma. Takashi et al. first reported four cases of duodenum-preserving total pancreatectomy, with or without preservation of the CBD and spleen, while conserving the splenic vessels in 2009.^26^ Tatsunosuke et al. documented a case of total parenchymal pancreatectomy preserving the duodenum, choledochus, and spleen for MD-IPMN, with no observed recurrence during a 30-month follow-up period.^27^ For MD-IPMN, DSP-TP is a valuable organ-preserving technique that provides significant radicality and is less invasive than classical total pancreatectomy. This procedure minimizes the impact on pancreatic disfunction and allows patients to maintain good nutritional status after surgery.

The magnified surgical field provided by the laparoscopic system facilitates the meticulous anatomical dissection of the vascular arcades in the pancreatic head and splenic vessels, thereby improving the success rate of DSP-TP.^28^ Our current multimedia research on LDSP-STP/TP for MD-IPMN combines the DPPHR technique and the Kimura technique, offering patient selection strategies and adhering to the principle of restricting excisional surgery to the target organ while maximizing the preservation of physiological function. The technical challenge of LDSP-STP/TP lies in controlling intraoperative bleeding from the vascular arcades in the pancreatic head, with vascular tear owing to excessive traction or incomplete coagulation using the ultrasonic scalpel being two important causes of bleeding. During hemostasis, smaller branches from the vascular arcades to the pancreas can be coagulated using the ultrasonic scalpel, while larger branches can be ligated or occluded using Hem-o-lock clips. Direct bipolar electrocoagulation or suture hemostasis may potentially damage the main trunk of the vascular arcades. In this study, LDSP-STP/TP was successfully performed on six patients without conversion to open surgery, and the posterior arcade of the pancreatic head and the AIPDA were completely preserved. Adequate blood supply to the duodenum was observed intraoperatively, and there were no cases of duodenal, CBD, or splenic ischemia postoperatively. The patients were able to resume eating within 3–5 days after surgery. These findings suggest that LDSP-STP/TP is feasible in centers with mature laparoscopic techniques in pancreatic surgery.

However, for the management of MD-IPMN, besides the technical feasibility, what is more important is how to determine the benign or malignant nature of the tumor and the extent of its spread.^29^ On the basis of existing guidelines and diagnostic techniques, we propose a patient selection strategy for LDSP-STP in treating MD-IPMN. Patients should exhibit one or more worrisome features to justify prophylactic surgery. The presence of high-risk stigmata strongly suggests malignant potential, and such patients should undergo oncological resection^6,8^ Postoperative glycemic control is challenging in patients undergoing TP, and patients with MD-IPMN always have a favorable postoperative survival. Preserving a portion of the pancreatic tail can help patients achieve better postoperative glycemic control. Previous studies have also demonstrated an improvement in patients’ quality of life with STP.^30,31^ In this study, except for the patient who underwent LDSP-TP, the remaining five patients who underwent LDSP-STP achieved good long-term blood glycemic control. However, the challenge with adopting the STP strategy for MD-IPMN lies in assessing the appropriate length of the preserved pancreatic tail and minimizing the risk of residual pancreatic tail harboring high-grade dysplasia or early invasive cancer. According to the current IPMN resection policies, in patients without high-risk stigmata, the majority of resected IPMNs do not contain high-grade dysplasia or invasive cancer. Therefore, for this subset of patients with IPMN, organ-preserving or parenchyma-sparing pancreatectomy appears to be oncologically safe^32–34^ Intraoperative ultrasound can guide the surgeon in selecting a segment of the pancreatic tail with a normal MPD diameter and no dilation as the site of pancreatic transection. In centers where the equipment conditions permit, intraoperative pancreatoscope can assist in detecting skip lesions^16–18^ Importantly, even if intraoperative frozen section results do not indicate the presence of high-grade dysplasia or invasive cancer, LDSP-STP/TP should be avoided in patients with a family history of pancreatic cancer or germline mutations to minimize the risk of residual pancreatic tissue developing into invasive cancer^12,13^

## Conclusions

LDSP-STP/TP demonstrates technical feasibility and safety. It allows for the selective resection of the affected pancreatic parenchyma, thereby minimizing the impact of pancreatic functional impairment. The proposed patient selection strategy is expected to reduce the risk of residual pancreatic tissue developing into invasive cancer. However, it is crucial to validate this strategy through long-term prospective observations.

## Supplementary Information

Below is the link to the electronic supplementary material.

Laparoscopic duodenum and spleen-preserving total pancreatectomy for main duct intraductal papillary mucinous neoplasms
Supplementary file1 (MP4 311623 KB)
